# Forgetting to take antiseizure medications is associated with focal to bilateral tonic-clonic seizures, as revealed by a cross-sectional study

**DOI:** 10.1371/journal.pone.0240082

**Published:** 2020-10-01

**Authors:** Hime Suzuki, Nobuhiro Mikuni, Hirofumi Ohnishi, Rintaro Yokoyama, Rei Enatsu, Satoko Ochi

**Affiliations:** 1 Department of Neurosurgery, Sapporo Medical University, Sapporo, Japan; 2 Department of Public Health, Sapporo Medical University, Sapporo, Japan; University of Rome Tor Vergata, ITALY

## Abstract

**Objectives:**

To evaluate the effects of nonadherence to antiseizure medications (ASMs) and clinical characteristics on seizure control, we employed a prospective cohort cross-sectional study using self-reports and medical records of patients with epilepsy (PWEs).

**Methods:**

Eight hundred and fifty-five PWEs taking ASMs were enrolled from fourteen collaborative outpatient clinics from January 2018 to March 2019. Questions from the Morisky Medication Adherence Scale were used as adherence self-reports. If a PWE’s questionnaire indicated that they had missed doses of their ASMs, outpatient physicians asked them directly about the details of their compliance, including the timing of intentionally or unintentionally missed doses. The association between lack of seizure control and utilization outcomes, such as missed doses, demographics, and clinical characteristics of the PWEs, were assessed by univariate and multivariate analyses.

**Results:**

Multivariate analysis revealed that forgetting to take ASMs was associated with lack of seizure control and the existence of focal to bilateral tonic–clonic seizures. Dementia, younger age, use of three or more antiepileptic agents, and living in a one-person household were associated with the risk of forgetting to take ASMs.

**Significance:**

For PWEs with poor drug management or a high incidence of missed doses of ASMs, efforts to improve adherence could facilitate better seizure control and decrease focal to bilateral tonic–clonic propagation.

## Introduction

Epilepsy is a chronic neurological disorder that is associated with elevated mortality and morbidity. The World Health Organization (WHO) estimates that about 50 million people around the world suffer from epilepsy, and up to 70% of patients with epilepsy (PWEs) could live seizure-free if properly diagnosed and treated with antiseizure medications (ASMs) [1]. Among PWEs, nonadherence to ASM treatment plans can result in status epilepticus [2, 3], hospital admission [4], mortality [5], and increased health care costs [6, 7]. The risk of hospital or emergency admission associated with epilepsy or non-febrile convulsions is 21% higher among nonadherers than adherers [8].

These data were reported prior to the International League Against Epilepsy (ILAE) 2017 operational classification of seizure types [9], and as yet there is no available evidence regarding the relationship between nonadherence and control of these seizure types. Focal to bilateral tonic–clonic seizure is defined as a special category of focal onset seizures in the ILAE 2017 classification of seizure types because of its common occurrence and importance [9]; it is one of the factors associated with epilepsy-related injuries and accidents [10]. To evaluate the risk of nonadherence to ASMs and clinical characteristics on lack of control of various seizure types including focal to bilateral tonic–clonic seizure, we employed a prospective cohort cross-sectional study using self-reports and medical records of PWEs.

## Materials and methods

We enrolled all consecutive PWEs taking ASMs, without *a priori* determination, suffering from focal or generalized onset epilepsy according to the ILAE 2017 classification of seizure types [9] from fourteen collaborating outpatient clinics from January 2018 to March 2019. All patients provided written informed consent before participating. If PWEs were 15 or under, informed consent was obtained from their parents. In the absence of parents of PWEs between 16 and 18 years old, grandparents or relatives who can judge whether or not to participate in this study, are allowed to substitute. PWEs who could not consent to study entry due to brain dysfunction such as severe dementia, or who could not participate in follow-up visits, or who changed treatments during the study were excluded. Dementia was diagnosed according to the Japanese translation of the Diagnostic and Statistical Manual of Mental Disorders (DSM–5) (The Japanese Society of Psychiatry and Neurology editorial supervision). This study was approved by the institutional ethics committee, and was performed in accordance with the ethical standards of the 1964 Declaration of Helsinki and its later amendments. This cross-sectional study protocol was approved by the Ethics Committee of Sapporo Medical University Hospital (No. 302–1199).

The questionnaires given to the participants included questions regarding adherence from the Morisky Medication Adherence Scale (Q 1. Do you ever forget to take your medicine?; Q 2. Are you careless at times about taking your medicine?; Q 3. When you feel better, do you sometimes stop taking your medicine?; Q 4. Sometimes if you feel worse when you take the medicine, do you stop taking it?). MMAS Research LLC USA confirmed that the scale used in the study is the "Morisky Medication Adherence Scale" and in the public domain [11]. The questionnaires also included questions on seizure symptoms such as cognitive, automatisms, and behavior arrest, which are common ictal semiologies of focal onset epilepsy [9, 12]. PWEs were asked to verify medication adherence and seizures with their caregivers. At the next outpatient examination, PWEs submitted their answers to the questionnaires after confirming their degree of adherence and seizures with their caregivers. If the participant responded “yes” to more than one question from the Morisky Medication Adherence Scale, the outpatient physicians directly asked them about their medication compliance (i.e., under 50%, 50–80%, over 80%) and the timing of intentionally and/or unintentionally missed doses (i.e., morning, noon, and evening). Medical records of the participants were obtained from the outpatient clinics. Variables abstracted from medical records included sex, age, seizure type [9], one-person household or not, diagnosis of dementia or not, etiology of epilepsy, frequency of seizures, and ASMs. Judging from the distribution of seizure frequency and clinical significance, we defined individuals experiencing more than one seizure a month as having poorly controlled seizures.

### Statistical analysis

Student's t-test and Fisher's exact probability test were used for the comparison between PWEs with well-controlled seizures and those with poorly controlled seizures. For tests that resulted in *p* < 0.05, simple logistic regression was used in the univariate analysis. Odds ratios (ORs) and 95% confidence intervals (CIs) were calculated for each model. Each item was then selected according to stepwise methods (model selection criterion, 0.10), and a multivariate analysis of all potential factors associated with poorly controlled seizures was performed. Age was classified into nine categories: 15 and under (pediatric patients), 16–19, 20–29, 30–39, 40–49, 50–59, 60–69, 70–79, and over 80, and ORs of each category were calculated with reference to the over-80 group. The Morisky score was calculated as total number of affirmative answers to four questions of the Morisky Medication Adherence Scale, and then classified into three categories (0, 1, and ≥2), and ORs of each category was calculated with reference to the group with a Morisky score of 0. Because the Morisky score is strongly related to the answer for each question on the Morisky Medication Adherence Scale, multivariate analysis using the Morisky score as a value of the Morisky Medication Adherence Scale was performed as “multivariate analysis model 1”, and multivariate analysis of answer of each question of the Morisky Medication Adherence Scale instead of the Morisky score was performed as “multivariate analysis model 2”. All statistical analyses were conducted using the SPSS software package (version 24.0, IBM Corp.), and *p* < 0.05 was considered to indicate statistical significance.

## Results

### Population and comparison of variables between well- and poorly controlled seizure groups

Our study population included 855 PWEs (male, 442; female, 413) ranging in age from 9 to 94 years (mean, 54.11 ± 20.86). Of those, 696 PWEs suffered from focal onset epilepsy and 159 suffered from generalized onset epilepsy. Among PWEs with focal onset epilepsy, 106 have focal aware seizures, 559 have focal impaired-awareness seizures, and 416 PWEs have focal to bilateral tonic-clonic seizures. Number of ASMs use was 1 (i.e. monotherapy) in 558 PWEs and 2 and more (i.e. polytherapy) in 297. Mean ASMs used per patient was 1.48. A total of 555 (64.9%) PWEs had well-controlled seizures, and 300 (35.1%) had poorly controlled seizures. Independent variables were compared between well- and poorly controlled seizure groups (Table 1).

**Table 1:** Comparison of variables between well and poorly controlled seizure groups.

Variables	Seizure control group		
	Well controlled (n = 555)	Poorly controlled (n = 300)	*p* value
Sex			**0.014**
Male/Female	304/251	138/162	
Age category			0.139
-15 years	5	6	
16–19	22	12	
20–29	64	33	
30–39	70	49	
40–49	63	21	
50–59	82	36	
60–69	107	55	
70–79	89	46	
80-	53	42	
Dementia	22	30	**<0.001**
One-person household(Yes)	73	42	0.729
Etiology			**0.001**
Brain tumor	67	34	
Cerebrovascular disease	188	78	
Inflammation	4	13	
Trauma	44	27	
Congenital abnormality	87	51	
Degenerative disease	32	32	
Others/Unknown	133	65	
Morisky score			**<0.001**
0	262	84	
1	158	102	
2	97	84	
3	32	16	
4	6	14	
Answer to Morisky Medication Adherence Scale questions			
Q1 (no/yes)	311/244	111/189	**<0.001**
Q2 (no/yes)	410/145	189/111	**0.001**
Q3 (no/yes)	524/31	276/24	0.170
Q4 (no/yes)	503/52	250/50	**0.002**
Seizure type			
Focal onset	424	272	**<0.001**
Generalized onset	131	28	
Number of ASMs use			
1	399	159	**<0.001**
2	132	80	
3	16	46	
4	8	10	
5	0	5	
Phenitoin (Yes/No)	44/511	16/284	0.164
Levetiracetam (Yes/No)	218/337	166/134	**<0.001**
Zonisamide (Yes/No)	106/449	47/253	0.225
Gabapentin (Yes/No)	3/552	10/290	**0.002**
Carbamazepine (Yes/No)	66/489	35/265	1.000
Sodium valproate (Yes/No)	145/410	61/239	0.065
Topiramate (Yes/No)	1/554	6/294	**0.009**
Lacosamide (Yes/No)	34/521	52/248	**<0.001**
Perampanel (Yes/No)	24/531	53/247	**<0.001**
Phenobarbital (Yes/No)	19/536	8/292	0.683
Clobazam (Yes/No)	21/534	18/282	0.169
Lamotrigine (Yes/No)	42/513	36/264	**0.035**
Clonazepam (Yes/No)	20/535	14/286	0.466

Significant p-values are in bold

ASMs: antiseizure medications

Variables such as sex; dementia; etiology; Morisky score; answers to Morisky Medication Adherence Scale questions (“yes” to Q1, Q2, and Q4); seizure type; number of total ASM use; and type of ASM such as levetiracetam, gabapentin, topiramate, lacosamide, perampanel, and lamotrigine showed statistically significant differences between the two groups. As for the ASMs, gabapentin, topiramate, perampanel, and clobazam were used only as ASM polytherapy due to restrictions related to Japanese insurance; consequently, use of these ASMs indicates the use of more than two total ASMs. The number of total ASMs used and the type of ASM (e.g., levetiracetam, lacosamide, and lamotrigine) were selected for further statistical analysis.

### Logistic regression analyses associated with poorly controlled seizures in all PWEs (Table 2)

**Table 2:** The univariate and multivariate logistic regression analyses associated with poorly controlled seizures in all PWEs.

	Univariate analysis		Multivariate analysis model 1		Multivariate analysis model 2	
Characteristics	Odds ratio (95% CI)	*p* value	Odds ratio (95% CI)	*p* value	Odds ratio (95% CI)	*p* value
Sex (Female)	1.42 (1.07–1.89)	**0.014**	1.45 (1.05–1.99)	**0.024**		
Age category						
≥80	1.00 (ref)	-	1.00 (ref)	-	1.00 (ref)	**-**
70–79	0.65 (0.38–1.12)	0.120	0.80 (0.44–1.44)	0.454	0.75 (0.41–1.40)	0.370
60–69	0.65 (0.39–1.09)	0.102	0.67 (0.37–1.20)	0.179	0.63 (0.34–1.16)	0.139
50–59	0.55 (0.32–0.97)	**0.040**	0.55 (0.29–1.04)	0.066	0.50 (0.25–0.99)	**0.047**
40–49	0.42 (0.22–0.80)	**0.008**	0.31 (0.14–0.65)	**0.002**	0.25 (0.11–0.56)	**0.001**
30–39	0.88 (0.51–1.52)	0.656	0.63 (0.32–1.22)	0.168	0.54 (0.26–1.11)	0.093
20–29	0.65 (0.36–1.17)	0.149	0.54 (0.27–1.07)	0.077	0.48 (0.22–1.02)	0.057
15–19	0.69 (0.31–1.55)	0.367	0.73 (0.30–1.80)	0.492	0.56 (0.21–1.52)	0.254
-15	1.51 (0.43–5.31)	0.517	3.86 (0.98–15.20)	0.053	3.41 (0.83–14.12)	0.090
Seizure Type (Focal onset)	3.00 (1.94–4.64)	**<0.001**	2.96 (1.82–4.83)	**<0.001**	3.41 (2.04–5.70)	**<0.001**
Etiology						
tumor	1.00 (ref)	-			1.00 (ref)	-
cerebrovascular	0.82 (0.50–1.33)	0.420			1.00 (0.58–1.74)	0.991
inflammatory	6.40 (1.94–21.14)	**0.002**			7.66 (2.02–29.14)	**0.003**
trauma	1.21 (0.64–2.28)	0.556			1.42 (0.69–2.90)	0.338
congenital	1.16 (0.67–1.98)	0.599			1.39 (0.73–2.65)	0.314
degenerative	1.97 (1.04–3.74)	**0.038**			1.79 (0.85–3.77)	0.128
others	0.96 (0.58–1.60)	0.885			1.34 (0.73–2.47)	0.341
Morisky score						
0	1.00 (ref)	-	1.00 (ref)	-	-	-
1	2.01 (1.42–2.86)	**<0.001**	1.68 (1.14–2.47)	**0.009**	-	-
≥2	2.70 (1.84–3.96)	**0.001**	2.45 (1.65–3.63)	**<0.001**	-	-
Morisky questions						
Q1 (Yes)	2.17 (1.63–2.90)	**<0.001**	-	**-**	1.99 (1.41–2.81)	**<0.001**
Q2 (Yes)	1.66 (1.30–2.25)	**0.001**	-	-		
Q3 (Yes)	1.47 (0.85–2.55)	0.172	-	-		
Q4 (Yes)	1.94 (1.28–2.94)	**0.002**	-	**-**	1.85 (1.13–3.03)	**0.015**
Dementia	2.69 (1.52–4.76)	**0.001**	1.90 (0.98–3.67)	0.056		
One-person household (Yes)	1.08 (0.71–1.62)	0.729				
Number of ASMs use						
1	1.00 (ref)	**-**	1.00 (ref)		1.00 (ref)	**-**
2	1.52 (1.09–2.12)	**0.014**	1.49 (1.03–2.18)	**0.036**	1.61 (1.10–2.36)	**0.014**
≥ 3	6.38 (3.84–10.59)	**<0.001**	5.84 (3.29–10.36)	**<0.001**	5.22 (2.90–9.40)	**<0.001**
Levetiracetam (Yes/No)	1.92 (1.44–2.55)	**<0.001**	1.71 (1.25–2.35)	**0.001**	1.79 (1.30–2.47)	**<0.001**
Lacosamide (Yes/No)	3.21 (2.03–5.08)	**<0.001**	2.01 (1.20–3.39)	**0.008**	2.09 (1.23–3.56)	**0.006**
Lamotrigine (Yes/No)	1.67 (1.04–2.66)	**0.033**				

Significant p-values are in bold

CI: confidence interval, Morisky questions: Morisky Medication Adherence Scale questions

ASMs: antiseizure medications

Nagelkerke R square; 0.236 in Model 1, 0.247 in Model 2

In the univariate analysis, lack of seizure control was associated with being female, age category 50–59 (vs. ≧80), age category 40–49 (vs. ≧80), having focal onset seizures, etiology of inflammatory, etiology of degenerative, Morisky score of 1 (vs. 0), Morisky score of ≥2 (vs. 0), responding “yes” to Morisky Medication Adherence Scale questions Q1, Q2, and Q4, dementia, use of 2 antiepileptic agents (reference is use of 1 antiepileptic agent), use of ≥3 antiepileptic agents (reference is use of 1 antiepileptic agent), use of levetiracetam, use of lacosamide, and use of lamotrigine. In multivariate analysis model 1, nine items were selected according to stepwise methods: lack of seizure control was associated with being female, age category 40–49 (vs. ≧80), having focal onset seizures, Morisky score of 1 (vs. 0), Morisky score of ≥2 (vs. 0), use of 2 antiepileptic agents (reference is use of 1 antiepileptic agent), use of ≥3 antiepileptic agents (reference is use of 1 antiepileptic agent), use of levetiracetam, and use of lacosamide. In multivariate analysis model 2, ten items were selected according to stepwise methods: lack of seizure control was associated with age category 50–59 (vs. ≧80), age category 40–49 (vs. ≧80), having focal onset seizures, etiology of inflammatory, responding “yes” to Morisky Medication Adherence Scale questions Q1 and Q4, dementia, use of 2 antiepileptic agents (reference is use of 1 antiepileptic agent), use of ≥3 antiepileptic agents (reference is use of 1 antiepileptic agent), use of levetiracetam, and use of lacosamide.

### Logistic regression analyses associated with focal to bilateral tonic–clonic seizure (Table 3)

**Table 3:** The univariate and multivariate logistic regression analyses associated with focal to bilateral tonic–clonic seizure.

	Univariate analysis		Multivariate analysis model 1		Multivariate analysis model 2	
Characteristics	Odds ratio (95% CI)	*p* value	Odds ratio (95% CI)	*p* value	Odds ratio (95% CI)	*p* value
Age (years)						
≥80	1.00 (ref)	-	1.00 (ref)	-	1.00 (ref)	-
70–79	1.65 (0.95–288)	0.077	1.46 (0.82–2.61)	0.201	1.27 (0.71–2.28)	0.423
60–69	1.68 (0.98–2.90)	0.060	1.41 (0.80–2.50)	0.239	1.23 (0.69–2.19)	0.483
50–59	1.66 (0.93–2.97)	0.089	1.26 (0.68–2.34)	0.466	0.99 (0.53–1.88)	0.985
40–49	2.09 (1.59–6.02)	**0.001**	2.38 (1.19–4.77)	**0.014**	1.98 (0.98–4.01)	0.058
30–39	3.88 (2.05–7.33)	**<0.001**	2.80 (1.43–5.48)	**<0.001**	2.26 (1.13–4.49)	**0.021**
20–29	4.13 (2.07–8.27)	**<0.001**	2.77 (1.33–5.77)	**0.006**	2.39 (1.14–5.03)	**0.021**
15–19	1.62 (0.63–4.15)	0.314	1.05 (0.40–2.78)	0.930	0.85 (0.32–2.30)	0.754
-15	0.90 (0.14–5.67)	0.911	0.74 (0.12–4.75)	0.751	0.62 (0.09–4.04)	0.614
Sex (Female)	0.83 (0.61–1.12)	0.227				
One-person household (Yes)	1.17 (0.75–1.82)	0.497				
Etiology						
tumor	1.00 (ref)					
cerebrovascular	0.89 (0.55–1.43)	0.617				
inflammatory	1.93 (0.57–6.50)	0.288				
trauma	1.26 (0.66–2.39)	0.485				
congenital	1.94 (1.05–3.57)	**0.034**				
degenerative	0.38 (0.19–0.75)	**0.005**				
others	1.19 (0.69–2.05)	0.531				
Dementia			0.41 (0.21–0.81)	**0.009**	0.38 (0.20–0.75)	**0.005**
Morisky score						
0	1.00 (ref)	**-**	1.00 (ref)	**-**	-	-
1	1.47 (1.03–2.11)	**0.036**	1.42 (0.98–2.06)	0.067	-	-
≥2	2.03 (1.39–2.97)	**<0.001**	1.93 (1.29–2.88)	**0.001**	-	-
Morisky questions						
Q1 (Yes)	2.22 (1.63–3.02)	**<0.001**	-	**-**	2.09 (1.50–2.92)	**<0.001**
Q2 (Yes)	1.54 (1.10–2.15)	**0.012**	-	-		
Q3 (Yes)	1.50 (0.80–2.83)	0.209	-	-		
Q4 (Yes)	0.63 (0.40–1.01)	0.054	-	-		
Number of ASMs use						
1	1.00 (ref)	-				
2	1.13 (0.79–1.62)	0.498				
≥ 3	1.94 (1.13–3.32)	**0.016**				
Levetiracetam (Yes/No)	1.03 (0.76–1.40)	0.833				
Lacosamide (Yes/No)	0.86 (0.53–1.39)	0.533				
Lamotrigine (Yes/No)	0.91 (0.54–1.53)	0.710				

Significant p-values are in bold

CI: confidence interval, Morisky questions: Morisky Medication Adherence Scale questions

ASMs: antiseizure medications

Nagelkerke R square; 0.091 in Model 1, 0.107 in Model 2

Focal onset seizure was significantly associated with the lack of seizure control; therefore, we further evaluated the risk of focal to bilateral tonic–clonic seizure.

In the univariate analysis, lack of seizure control was associated with age category 40–49 (vs. ≧80), age category 30–39 (vs. ≧80), age category 20–29 (vs. ≧80), etiology of congenital, etiology of degenerative, Morisky score of 1 (vs. 0), Morisky score of ≥2 (vs. 0), responding “yes” to Morisky Medication Adherence Scale questions Q1 and Q2, and use of ≥3 antiepileptic agents (reference is use of 1 antiepileptic agent). In multivariate analysis model 1, five items were selected according to stepwise methods: lack of seizure control was associated with age category 40–49 (vs. ≧80), age category 30–39 (vs. ≧80), age category 20–29 (vs. ≧80), dementia, and Morisky score of ≥2 (vs. 0). In multivariate analysis model 2, four items were selected according to stepwise methods: lack of seizure control was associated with age category 30–39 (vs. ≧80), age category 20–29 (vs. ≧80), dementia, responding “yes” to Morisky Medication Adherence Scale questions Q1.

### Logistic regression analyses associated with forgetting to take ASMs

Using the response to Q1 on the Morisky Medication Adherence Scale as the dependent variable, univariate and multivariate logistic regression analyses associated with forgetting to take ASMs are presented in Table 4.

**Table 4:** The univariate and multivariate logistic regression analyses associated with forgetting to take ASMs using the response to Q1 on the Morisky Medication Adherence Scale questions as the dependent variable.

	Univariate analysis		Multivariate analysis	
Characteristics	Odds ratio (95% CI)	*p* value	Odds ratio (95% CI)	*p* value
Dementia	1.15 (0.65–2.01)	0.634	2.74 (1.41–5.32)	**0.003**
Age category				
≥80	1.00 (ref)	**-**	1.00 (ref)	**-**
70–79	1.45 (0.83–2.55)	0.193	1.79 (0.97–3.30)	0.064
60–69	1.65 (0.96–2.83)	0.071	2.00 (1.09–3.67)	**0.026**
50–59	3.37 (1.90–5.98)	**<0.001**	4.46 (2.34–8.49)	**<0.001**
40–49	2.76 (1.49–5.11)	**0.001**	3.14 (1.58–6.27)	**<0.001**
30–39	4.23 (2.37–7.54)	**<0.001**	4.80 (2.48–9.29)	**<0.001**
20–29	5.09 (2.76–9.41)	**<0.001**	6.12 (3.08–12.15)	**<0.001**
15–19	7.78 (3.14–19.26)	**<0.001**	11.25 (4.34–29.20)	**<0.001**
-15	2.87 (0.81–10.19)	0.103	4.60 (1.25–16.85)	**0.021**
One-person household (Yes)	2.09 (1.39–3.16)	**0.001**	1.79 (1.16–2.77)	**0.009**
Number of ASMs use				
1	1.00 (ref)	**-**	1.00 (ref)	**-**
2	1.47 (1.07–2.03)	**0.017**	1.30 (0.93–1.82)	0.128
≥3	3.23 (1.94–5.36)	**<0.001**	2.46 (1.44–4.20)	**0.001**
Sex (Female)	1.12 (0.85–1.46)	0.424		

Significant p-values are in bold

CI: confidence interval, ASMs: antiseizure medications

Nagelkerke R square; 0.146

In the univariate analysis, responding “yes” to Q1 on the Morisky Medication Adherence Scale was associated with age category 50–59 (vs. ≧80), age category 40–49 (vs. ≧80), age category 30–39 (vs. ≧80), age category 20–29 (vs. ≧80), age category 15–19 (vs. ≧80), living in a one-person household, use of 2 antiepileptic agents (reference is use of 1 antiepileptic agent), and use of ≥3 antiepileptic agents (reference is use of 1 antiepileptic agent).

In the multivariate analysis, ten items were selected according to stepwise methods: responding “yes” to Q1 of the Morisky Medication Adherence Scale was associated with dementia, age category 60–69(vs. ≧80), age category 50–59 (vs. ≧80), age category 40–49 (vs. ≧80), age category 30–39 (vs. ≧80), age category 20–29 (vs. ≧80), age category 15–19(vs. ≧80), age category –15 (vs. ≧80), living in a one-person household, and use of ≥3 antiepileptic agents (reference is use of 1 antiepileptic agent).

### Other outcomes from the questionnaire

The frequencies of focal impaired-awareness seizures from the questionnaire are shown in Table 5. Number of PWEs with each seizure symptom was as follows: 442 for cognitive, 155 for automatisms, and 394 for behavioral arrest.

**Table 5:** The frequency of focal impaired-awareness seizure symptoms as outcome of questionnaires.

Type of symptom	Frequency of seizure symptoms			
	1–2 times per month	1–2 times per week	More than 3 times per week	Total (number)
Cognitive	263	117	62	442
Automatisms	98	33	24	155
Behavioral arrest	235	97	62	394

ASM compliance rates of 434 people who responded “yes” to Q1 of the Morisky Medication Adherence Scale were the following: 8 PWEs took ASMs less than 50% as often as scheduled; 39 PWEs took ASMs 50–80% as often as scheduled; and 387 PWEs took ASMs more than 80% as often as scheduled. The timing of missed doses (intentional or unintentional) is shown in Table 6.

**Table 6:** Number of missed doses by time of day according to unintentional or intentional reason of the PWEs.

	Unintentional (n = 411)	Intentional (n = 96)
Morning	111	17
Noon	10	0
Evening	231	64
Before sleep	2	0
Morning and evening	52	13
Morning, noon, and evening	5	2

## Discussion

Our study presents the first evidence regarding the relationship between adherence to ASMs and control of seizure type since the ILAE 2017 operational classification of seizure types became available. Using information obtained from PWEs via questionnaires, we found that forgetting to take ASMs is associated with not only a lack of seizure control, but also with having a focal to bilateral tonic–clonic seizure. Furthermore, we identified that dementia, younger age, use of three or more antiepileptic agents, and living in a one-person household are associated with the risk of forgetting to take ASMs.

A previous study reported that nonadherence varied between 26% and 79%, with the wide variability between studies likely resulting from different definitions of adherence and different cutoff values for nonadherence [13]. To evaluate adherence, several methods can be used, including self-reported information, measurement of serum drug levels, and tracking data from pharmacy prescription databases and electronic bottle tops. Studies using prescription tracking data or electronic caps provide more detailed information about treatment adherence. Those studies indicate that 50–80% of patients are defined as adherent when less stringent criteria are applied (e.g., taking the dose or at least having the medication on hand at least 80% of the time) [5, 6, 8, 14–16].

In our study, we applied the Morisky Medication Adherence Scale as the simplest method for measuring patient adherence to ASMs. Using a smaller sample size (50 PWEs), a previous study employed the same questions, but with the following modifications: adherence was classified as high if the answer to all four questions was “no,” moderate if the answer was “yes” for one or two questions, and low if the answer was “yes” for more than two questions. PWEs with moderate or low adherence were considered nonadherent. The authors found no difference in seizure control between groups with high adherence (21 patients) and those with moderate or low adherence (29 patients) [17]. Another study using the same Morisky Medication Adherence Scale reported that 66% of patients did not adhere to treatment, 60.5% were moderately adherent, and 5.7% had low adherence levels [18]. The authors reported that PWEs attributed nonadherence to forgetting doses (47.5%), lack of time to take ASMs (39.2%), worsening symptoms (9.0%), and symptom improvement (8.5%). In the present study, Morisky scores of ≥2 (using Morisky scores of 0 as a reference) and responding “yes” to Q1 on the Morisky Medication Adherence Scale (forgetting to take the medicine) was strongly associated with lack of seizure control and the existence of focal to bilateral tonic–clonic seizures.

If a PWE answered “yes” to one or more question on the questionnaires, outpatient physicians directly asked them about their ASM compliance (under 50%, 50–80%, over 80%), and the timing of intentionally and/or unintentionally missed doses. Among 433 PWEs who answered “yes” to Q1 (forgetting to take the medicine), 387 PWEs took the scheduled dose at least 80% of the time. Forgetting the evening dosing (unintentionally for 231 PWEs and intentionally for 64 PWEs) was the most common reason. Surprisingly, 26 PWEs who unintentionally missed dosing suffered from seizures on the same day or next day. Because all PWEs in this study have a two to four daily dosing regimen for medication, the decrease in plasma concentration of ASMs to an insufficient level following missed dosing may lead to occurrence of a seizure. Dosing frequency with once-daily dosing relative to more frequent (two to four times) daily dosing regimens facilitates adherence for chronic cardiovascular drugs, as well as antihypertensive drugs. Once-daily dosing of ASM monotherapy would likely improve adherence [18–20].

Using multivariate analysis, forgetting to take ASMs was associated with lack of seizure control and the occurrence of focal to bilateral tonic–clonic seizures; furthermore, forgetting to take ASMs was significantly associated with dementia, younger age, living in a one-person household, and use of three or more ASMs. Younger age, male sex, lower educational attainment, employment status (unemployed), uncontrolled seizure, and lower socioeconomic status in children are all factors associated with ASM nonadherence [13, 21–23], although dementia and living in a one-person household were evaluated as demographic factors for the first time in this study. Dementia was not a significant predictor in the univariate analysis but became significant in the multivariate analysis, indicating that other value of age associated with dementia was finally revised by multivariate analysis. Although PWEs suffering from severe dementia are excluded in our study, PWEs with poor drug management would benefit most from interventions that enhance adherence.

The responses to the questionnaires were confirmed at the next outpatient examination, after verification by their caregivers. Among patients with focal impaired-awareness seizures, the incidence of seizure symptoms as outcome of questionnaires was as follows: 64% for cognitive, 22% for automatisms, and 57% for behavioral arrest. PWEs with focal impaired awareness seizures are often not aware of their seizures; thus, participating in this study and responding to the questionnaire could inform the PWEs and their caregivers about the incidence of these seizures. Furthermore, caregivers would have an opportunity to understand the actual situation of impaired-awareness seizure and could care for the patient more safely and effectively.

Limitations of our methodology should be considered. The first limitation is bias from population heterogeneity; however, the rate of responding “yes” to more than one question (missing any dose) in this study (60%) was similar to the rate reported by previous studies (58–66%) using the Morisky Medication Adherence Scale [21, 24–26]. Methodological problems using self-reported information should also be considered. We asked the PWEs to confirm the outcome with their caregivers before submission, but there are patients who do not have caregivers or do not have a relationship with their caregivers. There is likely to be a gap between patient-reported and caregiver-reported answers, and it may be difficult for young or aged people to respond to the questionnaire.

Several other factors should be taken into account in our cross-sectional study. Significant comorbidity and drugs other than ASMs could influence seizure control and adherence to ASMs. Depressive symptoms are significantly correlated with seizure frequency and the number of antiepileptic drugs [27]. Mean duration of both epilepsy and depressive symptoms at the enrolment date may affect adherence. In this study, we did not investigate socioeconomic factors that could affect adherence to ASMs, such as educational background, employment, occupation, economic indicators (e.g., income), family structure, social support, and constant support for taking medication. Plasma concentrations of ASMs, as well as other treatments for epilepsy such as vagus nerve stimulation and dietary adjustments, could be correlated with seizure control.

Educational intervention such as sharing information between caregivers and PWEs with poor drug management and/or a high incidence of missed doses of ASMs could improve adherence, leading to reduction of seizures and decreasing focal to bilateral tonic–clonic propagation. Missed ASM doses have been reported as a risk factor for seizure-related motor vehicle crashes, hospitalizations, emergency department visits, fractures, and mortality [4, 5, 28]. In our study, 26 PWEs who unintentionally missed dosing suffered from seizures on the same day or next day, resulting in acute hospitalizations. Because our cross-sectional study did not follow outcomes, further studies to assess both adherence and control of seizures, taking into account dosing method and patient instruction, are necessary to clarify the clinical usefulness of our methods.

## Supporting information

S1 Data (XLSX)
